# Circular RNA circHSPA8 Aggravates Metastasis by Acting as a Competitive Inhibitor of miR‐195‐5p to Upregulate WNT3A Expression in Breast Cancer

**DOI:** 10.1111/jcmm.70499

**Published:** 2025-03-18

**Authors:** Zhuoying Han, Xiaojuan Yu, Chenlong Wang, Xiaoyu Song, Xiaomin Zhong, Renhua Guo, Weiyong Yu, Chao Luo

**Affiliations:** ^1^ Department of Central Laboratory The Affiliated Huaian No. 1 People's Hospital, Nanjing Medical University Huai'an China; ^2^ Department of Clinical Oncology The Affiliated Huaian No. 1 People's Hospital, Nanjing Medical University Huai'an China; ^3^ Department of Clinical Oncology The First Affiliated Hospital of Nanjing Medical University Nanjing China; ^4^ Biological Sample Bank The Affiliated Huaian No. 1 People's Hospital, Nanjing Medical University Huai'an China

**Keywords:** breast cancer, circHSPA8, circRNA, metastasis, miR‐195‐5p

## Abstract

Circular RNA (circRNA) plays a vital role in the tumorigenicity and progression of cancer by regulating various biological behaviours. It acts as a microRNA sponge, disrupting transcription and the abnormal expression of oncogenes. Hsa_circ_0024715, a circRNA generated from cyclization at specific sites of the *HSPA8* gene, has been found to be highly expressed in breast cancer (BC) tissue based on non‐coding RNA high‐throughput sequencing. However, its functions remain poorly understood. In this study, we performed qPCR to evaluate the expression of circHSPA8 in BC tissues. Survival analysis in a prospective cohort revealed that high expression of circHSPA8 is associated with poor prognosis and lymphoid node metastasis. Overexpression of circHSPA8 in MCF‐7 cells significantly enhanced their proliferative and invasive abilities, whereas knockdown of circHSPA8 in MDA‐MB‐231 cells significantly reduced their proliferative and invasive abilities. We found that circHSPA8 can promote epithelial–mesenchymal transition (EMT) in BC cells, primarily by upregulating the expression of *WNT3A*. This process depends on the sponging and inhibition of miR‐195‐5p, which suppresses the proliferation, invasion, and metastasis of BC cells. In vivo experiments further confirmed that circHSPA8 can promote the intravasation and extravasation of BC cells as well as the formation of metastatic lesions in the lungs. In summary, these data demonstrate that circHSPA8 promotes EMT by acting as a competitive inhibitor of miR‐195‐5p to upregulate the expression of *WNT3A* in BC, suggesting that dysregulation of circRNA in BC might be a pathological factor and potential therapeutic target.

## 1 Introduction

Breast cancer (BC) is the second most common malignant tumour in women, often occurring in younger women [1]. Advances in targeted and multidisciplinary therapies have reduced the high incidence and mortality in end‐stage patients, particularly those with aggressive forms such as triple‐negative BC [2, 3]. Therefore, further investigation of the tumour biology in BC is needed to identify novel potential therapeutic targets.

Circular RNA (circRNA) molecules play an important role in regulating various biological processes in tumours, especially tumour growth, cellular proliferation, apoptosis, therapeutic resistance, and metastasis [4, 5]. These molecules can repress the downregulation effect of microRNA on specific mRNA by acting as a sponge and binding competitively to microRNA molecules, which has been deemed a predominant mechanism involved in the transcriptional regulation effect of circRNA [6, 7, 8, 9]. The biogenesis of circRNAs may involve a co‐transcriptional product accompanied by traditional linear splices dependent on short intronic repeat sequences. We identified a circRNA molecule, highly expressed in BC tissues according to CircRNA Array analysis. Circ_hsa_0024715, located in the transcript region of *HSPA8* on chromosome 11, was noted. A previous study revealed that increased *HSPA8* expression in patients with BC is independently associated with a significant shortening of OS [10]. While the expression and regulatory mechanisms of *HSPA8* in BC remain unclear, microarray data reveal higher circHSPA8 expression in cancerous tissues. These data suggest that aberrant circularisation of HSPA8 may be involved in tumour biology and influence the dysfunction of downstream oncogenes. We further explored the potential of miRNAs to bind to circHSPA8, miR‐195‐5p, and the target genes of *WNT3A* according to the online website for bioinformatics prediction (TargetScan).

We verified bioinformatics predictions through in vitro experiments, finding a negative correlation between circHSPA8 expression and patient survival via q‐PCR. Unexpectedly, circHSPA8 transcription was higher in MDA‐MB‐231 cells than in MCF‐7 cells, which negated miR‐195‐5p's inhibition and upregulated WNT3A expression. This imbalance in circHSPA8 regulation may worsen tumour behaviour due to MDA‐MB‐231 cells' more aggressive invasion and metastasis compared to MCF‐7 cells. In conclusion, we discovered a previously unreported circRNA involved in the tumour biology of highly invasive BC cells.

## 2 Materials and Methods

### 2.1 Tissue Samples

Tumour tissues and adjacent normal breast tissues were collected from 153 BC patients who were undergoing radical surgery for BC at the Department of Breast Surgery, the Affiliated Huai'an No. 1 People's Hospital of Nanjing Medical University, from December 2012 to December 2015. ALL samples were stored in the biological sample bank and collected under the guidance of the HIPAA protocol and supervised by the ethics committee of Huai'an No. 1 People's Hospital. All patients were diagnosed with breast cancer by pathology after endoscopic biopsy before the operation and provided written informed consent before inclusion in this study. TNM stage classification complied with the TNM classification system of the International Union Against Cancer. We used Kaplan‐Meier method to draw the overall survival curve according to the relative expression of circHSPA8 and the cut‐off value for circHSPA8. This study was approved by the ethics committee of the Affiliated Huai'an No. 1 People's Hospital, Nanjing Medical University (KY‐2022‐005‐01).

### 2.2 CircRNA Array Analysis

Total RNAs from BC patients were collected for CircRNA analysis. As described previously [11], sample preparation was performed according to the standard protocols of RNA‐seq. Libraries were generated using the NEBNext Ultra RNA Library Prep Kit (New England Biolabs, Ipswich, MA, USA) for the Illumina system according to the manufacturer's instructions. Sequencing was conducted using the Illumina Hiseq XTEN platform. We defined the statistical criteria for selecting differentially expressed circRNAs using |fold changes| ≥ 1.0 with *p* values < 0.05.

### 2.3 Cell Culture

The human breast cancer cell lines MCF‐7 and MD‐MB‐231 were purchased from Zhongyuan Biotechnology Co. Ltd. (Beijing, China) and Xinyu Biotechnology Co. Ltd. (Shanghai, China). Cells were cultured in DMEM medium containing 10% FBS (invitrogen). In addition, the cultured medium was supplied with 1000 U/L penicillin and 1000 U/L streptomycin and maintained at 37°C in a humidified incubator containing 5% CO_2_ and 95% humidity.

### 2.4 CCK8

Cells were seeded at a density of 2000 cells per well into 96‐well plates and cultured for 1–4 days after treatment. A volume of 10 μL CCK‐8 solution was added to each well and incubated at 37°C for 2 h at 24 h, 48 h, and 72 h. The absorbance at 450 nm of the experimental wells was measured with an automatic microplate reader (Tecan, Switzerland).

### 2.5 Construction of Plasmid and Cell Transfection

The sequence of hsa_circ_0024715 (CircRNA HSPA8) was synthesised from Invitrogen (Qingke, Wuhan, China). The polymerase chain reaction (PCR) was performed using PCR Bestaq Master Mix (abm, Canada) with specific primers, and then the plasmid was extracted using the GeneJet plasmid MiniPrep Kit (Thermo Scientific). The plasmid was cloned into the pHB‐circBasic Vector by using HB‐infusion Master Mix (Jikai, Shanghai, China) according to the manufacturer's instructions. Similarly, the control vector was also constructed. Human_circ_0024715, shRNA_Human_circ_0024715, hsa‐miR‐195‐5p mimics and hsa‐miR‐NC were synthesised by and purchased from (GENERAL BIOL, Chuzhou, China). When cells were in logarithmic phase at 80% confluence, cells were collected and transfected with hsa_circ_0024715 over‐expression vector, shRNA_Human_circ_0024715, miR‐195‐5p mimics, inhibitor or corresponding negative control. Plasmid, or mimic were electroporated into 1 × 10^6^ cells (MCF‐7 or MDA‐MB‐231 cells) in 100 μL DMEM medium by using lipofectamine3000 according to the manufacturer's instructions. After transfection, the cells were incubated with the DMEM medium containing 10% FBS for 24–48 h under suitable conditions.

### 2.6 Flow Cytometric Analysis

For cell cycle analysis, collect BC cells and adjust the cell concentration to 1 × 10^6^ cells/mL. Take 1 mL of the single‐cell suspension, centrifuge, remove the supernatant, and add 70% cold ethanol for overnight fixation, storing at 4°C. According to the manufacturer's instructions, add PI/RNase A staining solution (KeyGEN BioTECH, China) and incubate at room temperature, protected from light, for 30 min. Measure cell cycle distribution using a CytoFLEX SRT flow cytometer (Beckman Coulter, USA). For apoptosis detection, collect cells after trypsin digestion, wash with pre‐cooled PBS, centrifuge, and discard the supernatant. Following the manufacturer's instructions, add Annexin V‐FITC Reagent and PI Reagent (Elabscience, China), vortex gently, incubate at room temperature, protected from light, for 15 min, and analyse using the CytoFLEX SRT flow cytometer.

### 2.7 RNA Preparation, Identification of circRNA Circularisation, Treatment With RNAse R, and PCR

RNA was isolated by TRIzol reagent (Invitrogen) according to the manufacturer's protocol. The circHSPA8 within breast cancer cells was selectively amplified utilizing divergent primers, which were designed based on the sequence information provided by circBase, specifically run through the back‐splice junction of hsa_circ_0024715. Subsequently, the amplified product underwent sequencing via the Sanger method to determine and map the back‐splice junction of hsa_circ_0024715, *t* which verifies the circularity of hsa_circ_0024715. Before extraction of total RNA for detection of expression of circHSPA8, the Rnase digestion reaction for 15 min at 37°C using 16 units RNaseR (Epicentre, Illumina, CA, USA) was performed in the cell lysis to delete the linear RNA and enrich circRNA molecules. Reverse transcription and qPCR were employed to analyse circHSPA8 expression and miRNA transcription. GAPDH, β‐actin, and RNU6‐1 were used as the internal references for circRNA, mRNA, and miRNA, respectively. The detecting primers were obtained from GENERAL BIOL Ltd. (Chuzhou, China). CircHSPA8 (divergent primers): Forward primer 5′‐TGCTGTGGACAAGAGTACGG‐3′, Reverse primer 5′‐AGACCAGCAATAGTTCCAGCA‐3′. CircHSPA8 (convergent primers): Forward primer 5′ GGTGGTTCTACTCGTATCCC 3′, Reverse primer 5′ ACATCCAAGAGCAGCAAATC 3′. miR‐195‐5p: Forward primer 5′ CGCAGCACAGAAATATTGGC 3′, Reverse primer 5′ CTCAACTGGTGTCGTGGAGTC 3′. U6: Forward primer 5′ GCTTCGGCAGCACATATACTAAAAT 3′, Reverse primer 5′ CGCTTCACGAATTTGCGTGTCAT 3′. GAPDH: Forward primer 5′ TATGATGACATCAAGAAGGTGGT 3′, Reverse primer 5′ TGTAGCCAAATTCGTTGTCATAC 3′. HSPA8: Forward primer 5′ AGCGTGCCATGACAAAGGAT 3′, Reverse primer 5′ CGTACTCTTGTCCACAGCAGA 3′. SNAIL: Forward primer 5′ CCTCCCTGTCAGATGAGGAC 3′, Reverse primer 5′ GCCTCCAAGGAAGAGACTGA 3′. VIM(vimentin): Forward primer 5′ TGACATTGAGATTGCCACCTACAG 3′, Reverse primer 5′ TCAACCGTCTTAATCAGAAGTGTCC 3′. CDH1(E‐cadherin): Forward primer 5′ GAGTGCCAACTGGACCATTCAGTA 3′, Reverse primer 5′ AGTCACCCACCTCTAAGGCCATC 3′. CDH2(N‐cadherin): Forward primer 5′ AGCACAGTGGCCACCTACAAAG 3′, Reverse primer 5′ CAGCTCCTGGCCCAGTTACA 3′.

### 2.8 Western Blot

Western Blot was performed using the primary antibodies: WNT3A (ab172612, Abcam, USA), E‐cadherin (#3195), N‐cadherin (#13116), Snail (#3879), Vimentin (#5741) (CST, USA), GAPDH (60004‐1‐Ig, Proteintech Group Inc., USA). The lysed cells were treated with lysis buffer for 10 min followed by centrifugation at 14,000 g for 1 min at 4°C. Protein concentration was determined using the BCA method (Biosharp, ShangHai, China). Protein samples were heat denatured at 100°C for 10 min. The samples were resolved by SDS‐PAGE and transferred onto PVDF membranes (Millipore, Bedford, MA). The membranes were blocked with 5% BSA (Thermo Fisher, CA, USA) in TBST buffer at room temperature for 2 h, and then the membrane was incubated at 4°C overnight with the primary antibody. We used a corresponding HRP‐labelled secondary antibody (Thermo Fisher, CA, USA) to incubate the membrane for 2 h at room temperature and then washed it 3 times with TBST buffer. The proteins were visualised using an enhanced chemiluminescent (Thermo Fisher, CA, USA) detection reagent (Tanon, Shanghai, China).

### 2.9 Clonogenic Assay

For the clonogenic assay, 5 × 10^2^ cells were seeded in a 6‐well plate for 2 weeks. Cell colonies were fixed with 4% PFA and stained with 0.1% crystal violet, and the stained colonies were photographed and then manually counted.

### 2.10 Wound‐Healing Assay

The wound‐healing assay was performed to evaluate the cell migration ability. Cells were seeded on six‐well plates in DMEM medium containing 10% FBS. After 24 h, straight lines were drawn by scraping the confluent cells with a 20 μL pipette tip and ruler. Then, the medium and floating cells were carefully removed, and the adherent cells were washed with PBS three times and continued to culture with serum‐free medium. Following a further 24 h incubation, the wound‐healing process was monitored under a phase‐contrast microscope, and the migration distance was measured, and representative images were obtained at 0 and 24 h, respectively.

### 2.11 Cell Migration and Invasion Ability Assays

Transwell assay used for the migration assay and invasion assay using the HTS transwell‐24 system (Corning, NY, USA). For the assay, the cells were cultured in serum‐free DMEM medium for 24 h, following which 100 μL of 5 × 10^4^ cells were seeded onto the upper chamber while the lower chamber was filled with 600 μL DMEM with 20% FBS. After incubation at 37°C for 24 h, cells on the bottom surface of the membrane were fixed and stained with 0.1% crystal violet. The cells on the upper surface of the inserts were then removed by scraping the inserts with a cotton swab. Migration was assessed by counting the number of penetrated cells in five random fields. Matrigel invasion assays were performed using Matrigel‐coated Transwell inserts following the same procedure described above [12].

### 2.12 Dual‐Luciferase Reporter Assay

A wild‐type or mut‐circHSPA8 fragment was constructed and inserted downstream of the luciferase reporter gene of the pMIR‐REPORT plasmid (AM5795, Thermo Scientific, Shanghai, China). We used Lipofectamine 3000 to transfect the reporter plasmid into breast cancer cells. We then co‐transfected the miR‐195‐5p inhibitor and mimic with the reporter gene into MCF‐7 and MDA‐MB‐231 cells, respectively. For the last step, we used the Dual‐Luciferase Reporter System Kit (E1910, Promega, USA) to detect firefly and renilla luciferase activity.

### 2.13 Assay of Tumour Cell Arrest in Lung and Extravasation

1 × 10^6^ CFSE‐labelled tumour cells were injected into mice via the tail vein. Lungs were harvested from mice 5 h and 24 h after tumour cell injection. Frozen sections were prepared and analysed by fluorescence microscopy. Fluorescent spots were counted from randomly chosen fields in the sections of each mouse.

### 2.14 Animal Experiments

Tumour cells were transfected with a luciferase tag, then the cells were untransfected or transfected with the indicated vectors or Lentivirus. NOD SCID mice were inoculated with MCF‐7 cells (2 × 10^6^ cells/mouse) and MDA‐MB‐231 cells (1 × 10^6^ cells/mouse). Tumour cells were injected into mice via the tail vein. 5 weeks after inoculation, the mice were sacrificed and lung metastases were analysed.

### 2.15 Statistical Analysis

All data was presented as mean ± SD. The Student *t* test was performed to analyse whether two experimental groups have a significant difference using *p* < 0.05 as the significant criterion. The ANOVA test was used for comparison of multiple groups over two. Survival analysis was performed using Kaplan–Meier curves and the log‐rank test in SPSS 23.0.

## 3 Results

### 3.1 Identification of the Circular Structure and Clinical Features of circHSPA8

**FIGURE 1:**
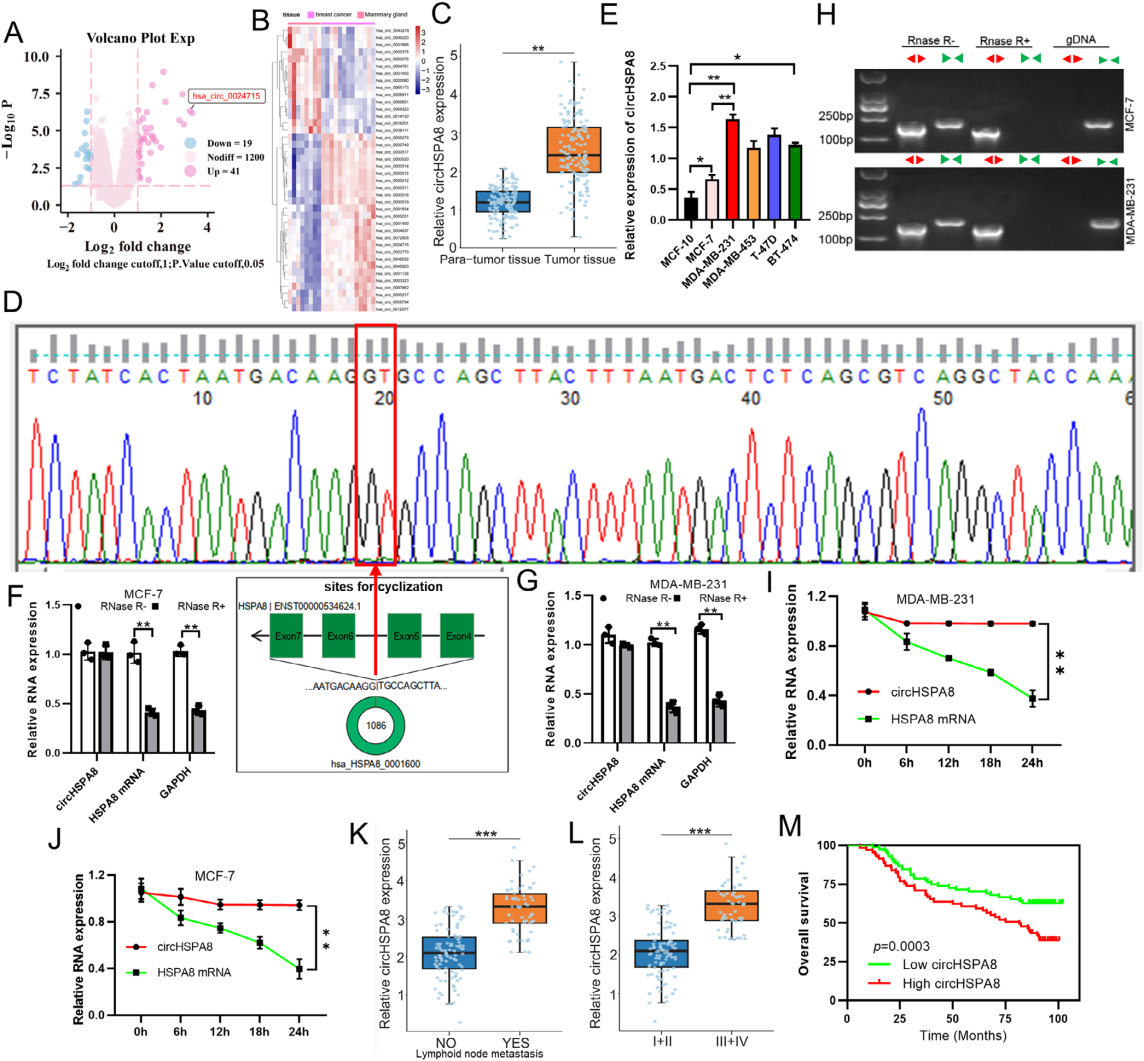
Identification of the circular structure and clinical features of circHSPA8. (A and B) We analysed the expression profiles of circular RNAs (circRNAs) in 13 breast cancer tissue samples and 8 normal breast tissue samples using circRNA microarray. Volcano plots were utilised to illustrate the differential expression levels and quantities of circRNAs (A), while cluster heatmaps were employed to further examine the patterns of differential circRNA expression (B). (C) The expression of circHSPA8 was analysed in tumour tissues and corresponding adjacent normal tissues from a cohort of 153 breast cancer patients using quantitative reverse transcription polymerase chain reaction (qRT‐PCR). (D) Divergent primers were designed based on the circHSPA8 cyclization site as documented in circBase and subsequently utilised for amplification via reverse transcription polymerase chain reaction (RT‐PCR). The resulting complementary DNA (cDNA) fragments were purified and subjected to Sanger sequencing to analyse the circHSPA8 cyclization site. (E) Quantitative real‐time PCR analysis of circHSPA8 expression levels in MCF‐7, MDA‐MB‐231, MDA‐MB‐453, T‐47D, and MCF‐10 cell lines. (F and G). In the quantitative reverse transcription PCR (qRT‐PCR) analysis, we employed specifically designed divergent and convergent primers to evaluate the RNase R resistance of circular HSPA8 in comparison to linear HSPA8 and GAPDH. (H) The levels of expression of the back‐spliced and canonical versions of HSPA8 were measured in BC cell cDNA and gDNA using PCR and agarose gel electrophoresis, with or without RNase R supplementation. (I and J). Investigate the stability of circular HSPA8 in breast cancer cells following exposure to actinomycin D, a transcriptional inhibitor. (K) Analysis of clinical samples to assess the differential expression of circHSPA8 in breast cancer patients with and without lymph node metastasis. (L) Analysis of clinical samples to assess the differential expression of circHSPA8 in breast cancer patients in different pathological stages. (M) Kaplan–Meier Survival Analysis of the association between CircHSPA8 expression in breast cancer and clinical prognosis. *p < 0.05, **p < 0.01, ***p < 0.001. (C, E–L) Data are from three independent experiments.

We performed circRNA microarray analysis of total RNA obtained from 13 clinical BC tissue samples and 8 adjacent normal tissue samples and constructed a circRNA profiling database. We detected 1260 distinct (circRNAs) and found that circHSPA8 was upregulated in BC (Figure 1A,B). To further confirm whether the expression level of circHSPA8 was high in BC tumours, we detected higher circHSPA8 expression in 153 BC samples than in adjacent normal samples using qRT‐PCR, which was consistent with the circRNA microarray analysis (Figure 1C). To ascertain the cyclic nature of circHSPA8 and confirm the consistency of its cyclization site with existing database records, we conducted sequencing based on the cyclization site sequence of hsa_circ_0024715 using divergent primers as documented in circBase. The sequencing results of the cDNA, amplified with divergent primers, revealed that the cyclization site of circHSPA8 corresponded with the circularization site listed in circBase, thereby confirming its circular structure formed in a back‐to‐back configuration (Figure 1D). Next, we confirmed higher levels of circHSPA8 in the MCF‐7, MDA‐MB‐231, MDA‐MB‐453, T‐47D, and BT‐474 cell lines than in MCF‐10 cells. MDA‐MB‐231 cells showed the highest expression level of circHSPA8, whereas MCF‐7 cells showed the lowest expression level (Figure 1E). Therefore, we used MCF‐7 and MDA‐MB‐231 BC cells to investigate the role of circHSPA8 in BC development. CircHSPA8 originates from *HSPA8*, which is located on chromosome 11. To explore whether the head‐to‐tail splicing products were derived from genomic rearrangements and trans‐splicing, we first performed a qRT‐PCR assay with specially designed divergent and convergent primers and discovered that circHSPA8, rather than linear‐HSPA8 or actin, could resist digestion by RNase R (Figure 1F,G). Moreover, we assessed the expression levels of the back‐spliced or canonical form of *HSPA8* in the cDNA and gDNA of BC cells, with or without RNase R supplementation, using PCR and agarose gel electrophoresis. We detected circHSPA8 in cDNA using divergent primers even after RNase R treatment. Conversely, the gDNA PCR products showed the opposite results. Additionally, the linear form of *HSPA8* could not be amplified using convergent primers, indicating that circHSPA8 was not attributable to genomic rearrangements or PCR artefacts (Figure 1H). Simultaneously, we also found that circHSPA8 is more stable than linear HSPA8 in BC cells treated with actinomycin D, a transcription inhibitor (Figure 1I,J). These results suggest that circHSPA8 is a suitable diagnostic or prognostic biomarker. Further analysis of clinical samples showed that the expression of circHSPA8 was significantly higher in patients with BC exhibiting lymph node metastasis than in those without lymph node metastasis (Figure 1K). Additionally, circHSPA8 expression increased with higher pathological stages (Figure 1L) and was negatively correlated with patient prognosis (Figure 1M).

### 3.2 CircHSPA8 Promotes the Proliferation, Migration, and Invasion Abilities of BC Cells In Vitro

**FIGURE 2:**
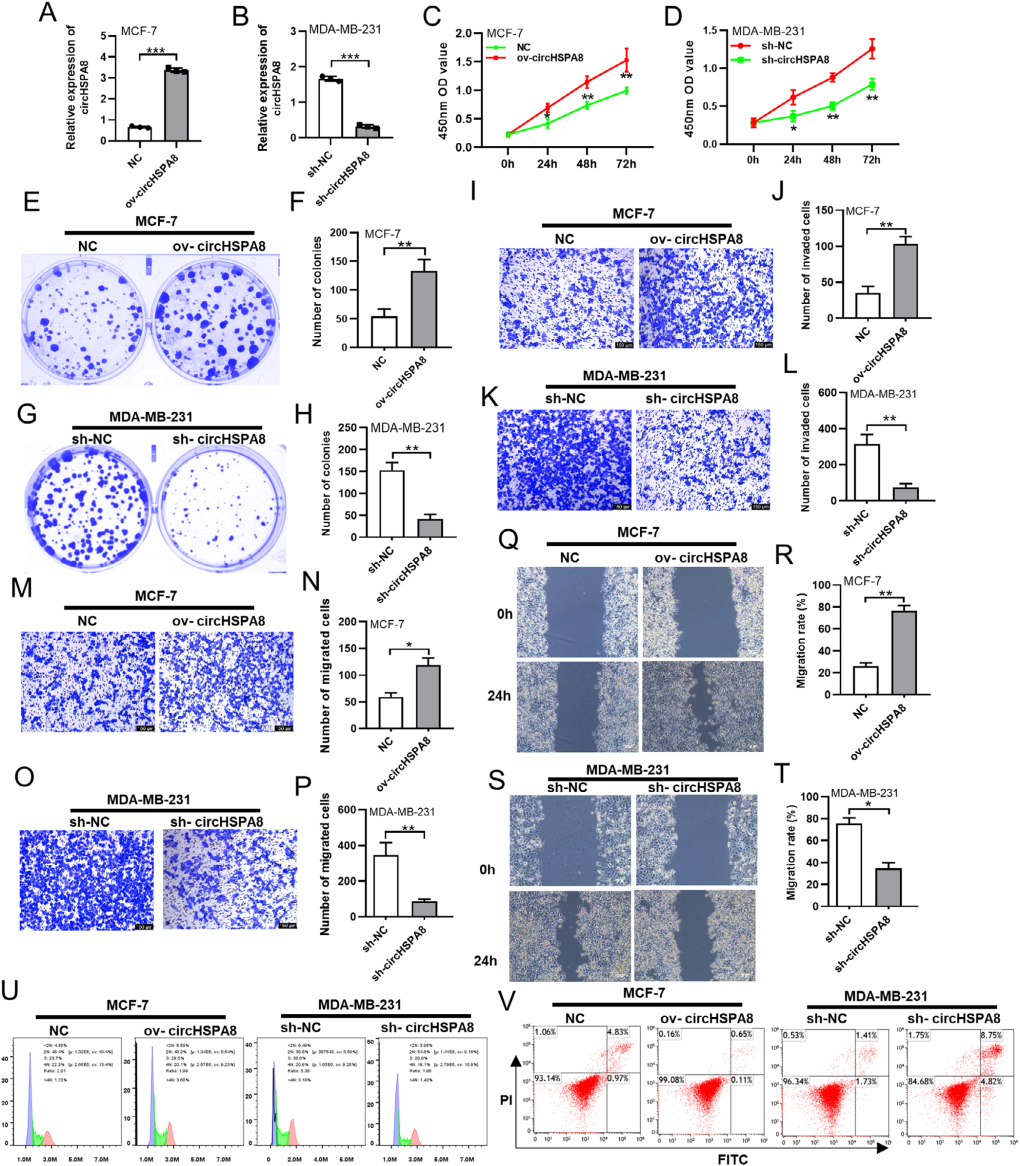
CircHSPA8 promotes the proliferation, migration, and invasion abilities of BC cells in vitro. (A and B) Overexpressed circHSPA8 in low‐metastatic MCF‐7 cells by transfecting with a circHSPA8 overexpression plasmid and knocked down circHSPA8 expression in highly metastatic MDA‐MB‐231 cells using lentiviral vectors. (C and D) The CCK8 assay was employed to evaluate the proliferative capacity of breast cancer cells following the overexpression or knockdown of circHSPA8 expression. (E–H) The colony‐forming capacity of breast cancer cells following circHSPA8 overexpression or knockdown was assessed using a plate cloning assay. (I–P) The invasion and migration capacity of breast cancer cells following circHSPA8 overexpression or knockdown was assessed using Transwell assay. (Q–T) The migration capacity of breast cancer cells following circHSPA8 overexpression or knockdown was assessed using wound healing assay. (U) DNA content of MCF‐7 cells and MDA‐MB‐231 cells was analysed by flow cytometry after overexpressed circHSPA8 or knocked down circHSPA8 expression respectively. (V) Annexin V‐FITC/PI double‐staining was used to assess the apoptosis of MCF‐7 cells and MDA‐MB‐231 cells after overexpressed circHSPA8 or knocked down circHSPA8 expression respectively. *p < 0.05, **p < 0.01, ***p < 0.001. Data are from three independent experiments.

Based on our previous confirmation that circHSPA8 expression is closely related to prognosis and lymph node metastasis in patients with breast cancer, we hypothesised that circHSPA8 expression promotes the malignant phenotype progression of BC cells. To confirm this hypothesis, we overexpressed circHSPA8 in low‐metastatic MCF‐7 cells by transfecting them with a circHSPA8 overexpression plasmid (Figure 2A) and knocked down circHSPA8 expression in highly metastatic MDA‐MB‐231 cells using lentiviral vectors (Figure 2B). In subsequent experiments, we assessed the proliferative ability of BC cells using CCK8 and colony formation assays, then evaluated cell migration capability using Transwell and wound healing assays. Our study demonstrated that the overexpression of circHSPA8 markedly enhanced the proliferation (Figure 2C), colony formation capacity (Figure 2E,F), invasion (Figure 2I,J), migration potential (Figure 2M,N,Q,R), and proliferative cycle (Figure 2U) of MCF‐7 cells, while concurrently inhibiting apoptotic processes (Figure 2V). Conversely, the suppression of circHSPA8 expression markedly reduced the proliferation (Figure 2D), colony formation capacity (Figure 2G,H), invasion (Figure 2K,L), and migration abilities (Figure 2O,P,S,T), as well as the proliferation cycle (Figure 2U) of MDA‐MB‐231 cells, while enhancing apoptotic activity (Figure 2V). These results further confirm that circHSPA8 expression promotes BC progression in BC cells.

### 3.3 CircHSPA8 Serves as a miRNA Sponge of miR‐195‐5p, Which Inhibits the Malignant Phenotype of BC Cells

**FIGURE 3:**
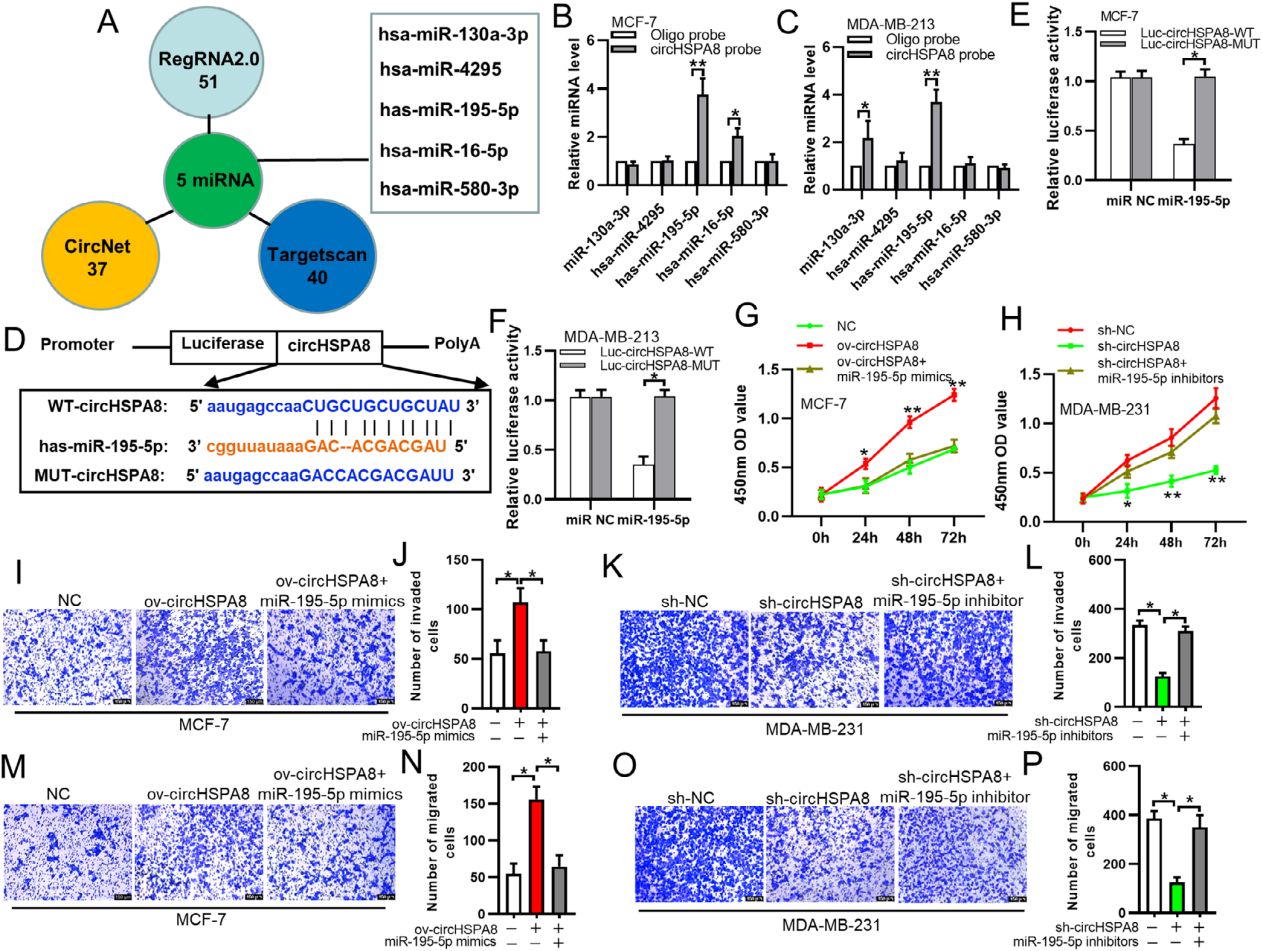
CircHSPA8 serves as a miRNA sponge of miR‐195‐5p, which inhibits the malignant phenotype of BC cells. (A) Through an analysis of three publicly available databases—RegRAN2.0, CircNet, and TargetScan—we identified the microRNA (miRNA) most likely to bind to circHSPA8. (B and C) Construct a targeted biotinylated circHSPA8 probe and induce the overexpression of biotinylated circHSPA8 in breast cancer (BC) cells. Subsequently, conduct a biotin pull‐down assay to isolate the biotinylated circHSPA8. Finally, employ quantitative reverse transcription PCR (qRT‐PCR) to quantify the expression levels of five specific microRNAs (miRNAs). (D–F) Through the design of a dual luciferase reporter gene plasmid and the execution of cell transfection experiments to assess dual luciferase activity, it was established that circHSPA8 and miR‐195‐5p exhibit direct binding interactions. (G and H) The proliferation of MCF‐7 cells with overexpressed circHSPA8 and the knockdown of circHSPA8 expression in MDA‐MB‐231 cells, which were transfected with miR‐195‐5p mimics and inhibitors, respectively, were assessed by the CCK8 assay. (I–P) The invasiveness and migration of MCF‐7 cells with overexpressed circHSPA8 and MDA‐MB‐231 cells with circHSPA8 knockdown, following transfection with miR‐195‐5p mimics and inhibitors, respectively, were evaluated using a transwell assay. *p < 0.05, **p < 0.01. Data are from three independent experiments.

CircRNAs act as miRNA sponges to regulate tumorigenesis and development. By sponging miRNAs, they indirectly regulate downstream genes associated with tumour occurrence and progression. Through predictions and analyses using public databases such as Circinteractome, CircNet, and RegRNA, we identified potential target miRNAs of circHSPA8. Based on conjugation scores, we screened five potential miRNAs for which circHSPA8 was most likely to be absorbed (Figure 3A). We designed specific biotin‐labelled circHSPA8 probes to confirm the potential miRNAs to which circHSPA8 binds. After overexpressing biotin‐labelled circHSPA8 in BC cells and performing a biotin pull‐down assay, we extracted biotin‐labelled circHSPA8 and detected the expression levels of the five miRNAs using qRT‐PCR. Our results confirmed the significant enrichment of circHSPA8 and miR‐195‐5p in the two BC cell lines, whereas other miRNAs did not show close interactions with circHSPA8 (Figure 3B,C). We conducted a dual‐luciferase reporter assay to determine the direct binding between circHSPA8 and miR‐195‐5p (Figure 3D). Wild‐type and mutant circHSPA8 fragments were inserted downstream of the luciferase reporter gene based on their complementary sequences. Plasmids containing the reporter gene and miR‐195‐5p mimic were co‐transfected into MCF‐7 and MDA‐MB‐231 cells. Compared to co‐transfection with control RNA, the luciferase activity of the wild‐type significantly decreased after miR‐195‐5p mimic transfection, whereas the luciferase activity of the mutant did not change significantly (Figure 3E,F). Therefore, we confirmed the direct interaction between circHSPA8 and miR‐195‐5p.

Next, we examined the proliferation, invasion, and migration abilities of MCF‐7 cells overexpressing circHSPA8 and transfected them with miR‐195‐5p mimics. Similarly, MDA‐MB‐231 cells were tested after knocking down circHSPA8 expression and transfecting them with miR‐195‐5p inhibitors. The CCK8 assay results showed that overexpression of circHSPA8 in MCF‐7 cells, followed by transfection with miR‐195‐5p mimics, abolished the promotive effect of circHSPA8 overexpression on cell proliferation (Figure 3G). In contrast, transfection with miR‐195‐5p inhibitors in circHSPA8‐knockdown MDA‐MB‐231 cells had the opposite effect (Figure 3F). Transwell assay results also indicated that overexpression of circHSPA8 in MCF‐7 cells, followed by transfection with miR‐195‐5p mimics, abolished the promotive effect of circHSPA8 overexpression on cell invasion and migration (Figure 3I,J,M,N). In contrast, transfection with miR‐195‐5p inhibitors in circHSPA8‐knockdown MDA‐MB‐231 cells restored their cell invasion and migration abilities (Figure 3K,L,O,P). In conclusion, circHSPA8 acts as a miR‐195‐5p sponge in BC cells, thereby promoting their malignant phenotype.

### 3.4 CircHSPA8 Promotes Epithelial–Mesenchymal Transition (EMT) in BC Cells

**FIGURE 4:**
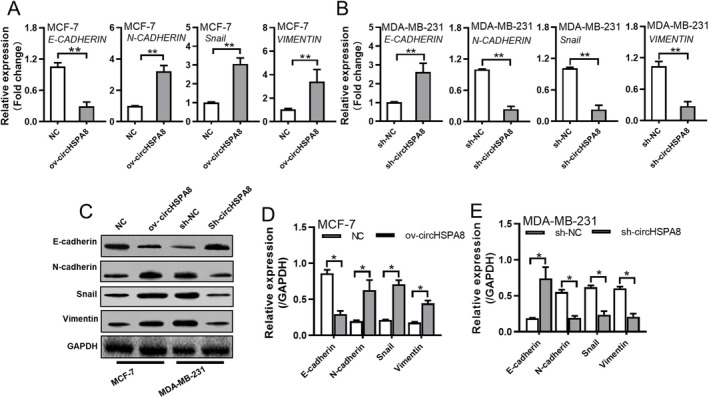
CircHSPA8 promotes EMT in BC cells. (A and B) Quantitative reverse transcription PCR (Q‐RT‐PCR) was employed to assess the expression of epithelial‐mesenchymal transition (EMT)‐related genes, including CDH1 (E‐cadherin), CDH2 (N‐cadherin), SNAIL, and VIM (Vimentin), following the overexpression of circHSPA8 in MCF‐7 cells (A) and the knockdown of circHSPA8 in MDA‐MB‐231 cells (B). (C) Western blot analysis was employed to assess the expression of the epithelial‐mesenchymal transition (EMT)‐related gene E‐cadherin following the overexpression of circHSPA8 in MCF‐7 cells and the knockdown of circHSPA8 in MDA‐MB‐231 cells. Additionally, the expression levels of N‐cadherin, SNAIL, and Vimentin were evaluated. (D and E) Statistical analysis results of EMT related genes protein levels. *p < 0.05, **p < 0.01. Data are from three independent experiments.

EMT is a crucial process through which tumour cells acquire invasive and metastatic capabilities. CircHSPA8 expression is closely associated with lymph node metastasis in patients, and in vitro experiments have indicated that CircHSPA8 expression promotes the invasion and migration abilities of BC cells. Therefore, we investigated the effect of circHSPA8 on EMT in BC cells. Firstly, we examined the impact of circHSPA8 overexpression and knockdown on the expression of EMT‐related markers, such as E‐cadherin, N‐cadherin, Snail1, and Vimentin, in BC cells using qRT‐PCR. The results showed that circHSPA8 overexpression in MCF‐7 cells significantly downregulated *CDH1* (E‐cadherin)expression and upregulated *CDH2* (N‐cadherin), *SNAIL*, and *VIM* (Vimentin)expression (Figure 4A). Conversely, knockdown of circHSPA8 in MDA‐MB‐231 cells significantly upregulated *E‐cadherin* expression and downregulated *N‐cadherin*, *Snail1*, and *Vimentin* expression (Figure 4B). We further validated these findings at the protein level using western blotting, which yielded results consistent with the qRT‐PCR data (Figure 4C–E). This suggests that circHSPA8 promotes lymph node metastasis in BC by regulating EMT‐related genes.

### 3.5 The circHSPA8‐miR‐195‐5p‐WNT3A Axis Promotes EMT and BC Progression

**FIGURE 5:**
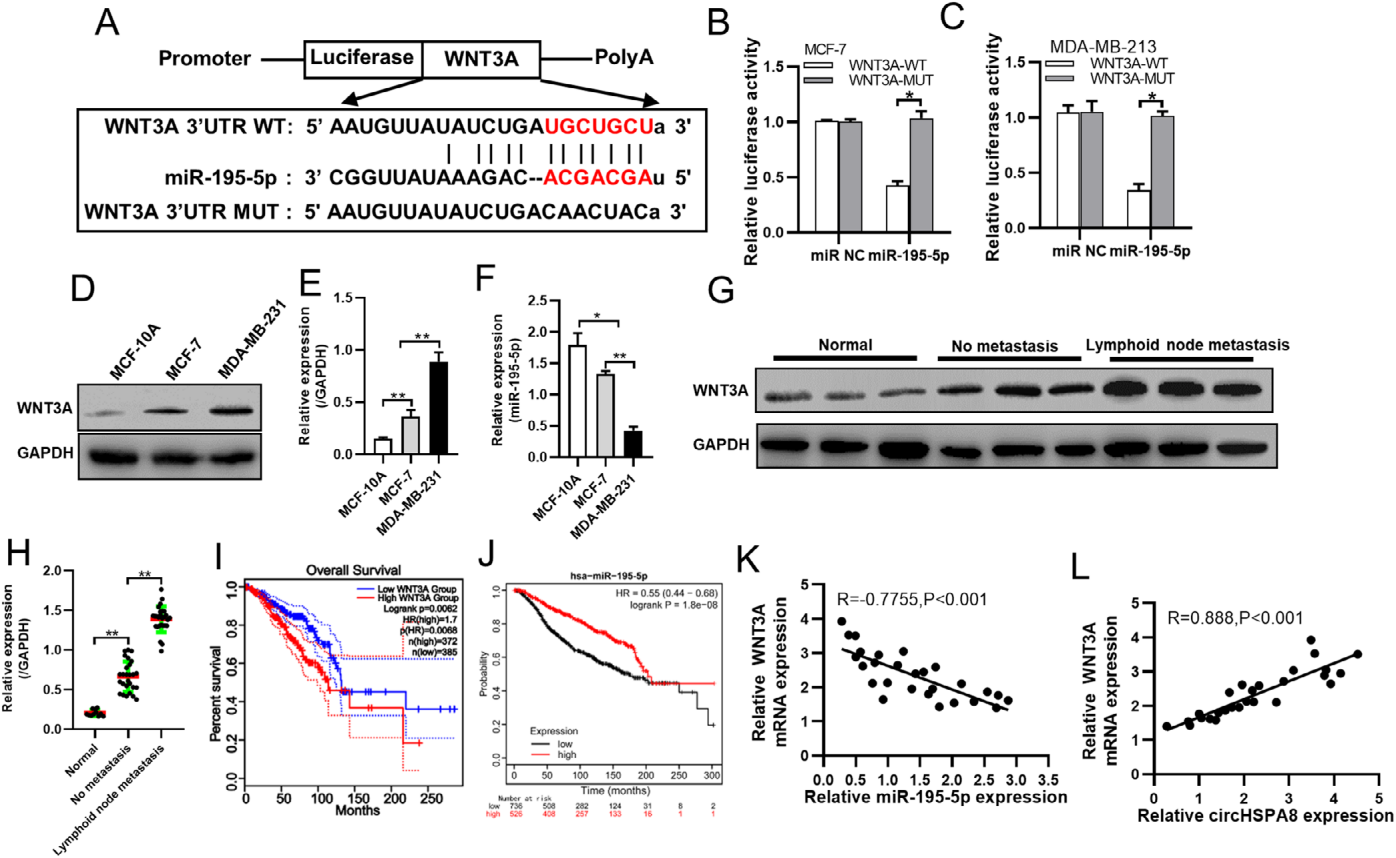
miR‐195‐5p inhibits WNT3A espression and suppresses the progression of BC by binding to WNT3A 3′UTR. (A) The predicted binding sites between miR‐195‐5p and WNT3A were presented. (B and C) The dual luciferase reporter assay is employed to validate the interaction between miR‐195‐5p and WNT3A. (D and E) The expression of WNT3A in both normal breast cells and breast cancer cells was analysed using Western blotting. (F) The expression levels of miR‐195‐5p in MCF‐10A, MCF‐7, and MDA‐MB‐231 cell lines were quantified using quantitative reverse transcription polymerase chain reaction (qRT‐PCR). (G and H) The expression of WNT3A in normal breast tissue, non‐metastatic breast cancer, and lymph node metastatic breast cancer tissues was assessed using Western blot analysis and subsequently quantified with ImageJ software. (I) Impact of WNT3A expression levels on overall survival in breast cancer patients: An analysis utilising mRNA sequencing data from GEPIA2 (http://gepia2.cancer‐pku.cn). (J) Impact of miR‐195‐5p expression levels on overall survival in breast cancer patients: An analysis using sequencing data from the Kaplan–Meier Plotter (https://kmplot.com/analysis). (K) The expression levels of WNT3A and miR‐195‐5p in 27 breast cancer cases were quantified using quantitative reverse transcription polymerase chain reaction (qRT‐PCR). Subsequently, the correlation between the expression levels of WNT3A and miR‐195‐5p was evaluated. (L) The expression levels of WNT3A and circHSPA8 in 27 breast cancer cases were quantified using quantitative reverse transcription polymerase chain reaction (qRT‐PCR). Subsequently, the correlation between the expression levels of WNT3A and circHSPA8 was evaluated. *p < 0.05, **p < 0.01. Data are from three independent experiments.

MiRNAs regulate gene expression by binding to the 3′ untranslated region (UTR) of target genes to inhibit translation or degrade mRNA, thus playing a role in tumorigenesis and cancer progression. Extensive research has indicated that miR‐195‐5p acts as a tumour suppressor in various cancers, which prompted us to investigate its role and mechanism of action in BC. Using public databases, we predicted potential target genes of miR‐195‐5p and identified *WNT3A* as a highly credible target. By aligning complementary sequences, we designed wild‐type and mutant fragments of *WNT3A* 3′ UTR and inserted them downstream of a luciferase reporter gene (Figure 5A). Dual‐luciferase reporter assays confirmed that miR‐195‐5p directly binds to the 3′ UTR of *WNT3A* in BC cells (Figure 5B,C).

Subsequently, we examined the expression of *WNT3A* and miR‐195‐5p in MCF‐7 and MDA‐MB‐231 cells. Results showed that WNT3A expression was significantly lower in MCF‐7 cells than in MDA‐MB‐231 cells (Figure 5D,E), whereas miR‐195‐5p expression was significantly higher in MCF‐7 cells (Figure 5F). Clinical tissue samples indicated that WNT3A expression was significantly higher in BC tissues than in adjacent normal tissues and was even higher in the tumour tissues of patients with lymph node metastasis than in those without (Figure 5G,H). Survival analysis of the TCGA database revealed that high *WNT3A* expression correlated with a poorer prognosis (Figure 5I), whereas higher miR‐195‐5p expression correlated with a better prognosis (Figure 5J). Clinical sample analysis showed a negative correlation between *WNT3A* and miR‐195‐5p expression levels (Figure 5K), and a positive correlation with circHSPA8 expression (Figure 5L). These results led us to hypothesise that circHSPA8 promotes EMT in BC via the miR‐195‐5p‐WNT3A axis.

**FIGURE 6:**
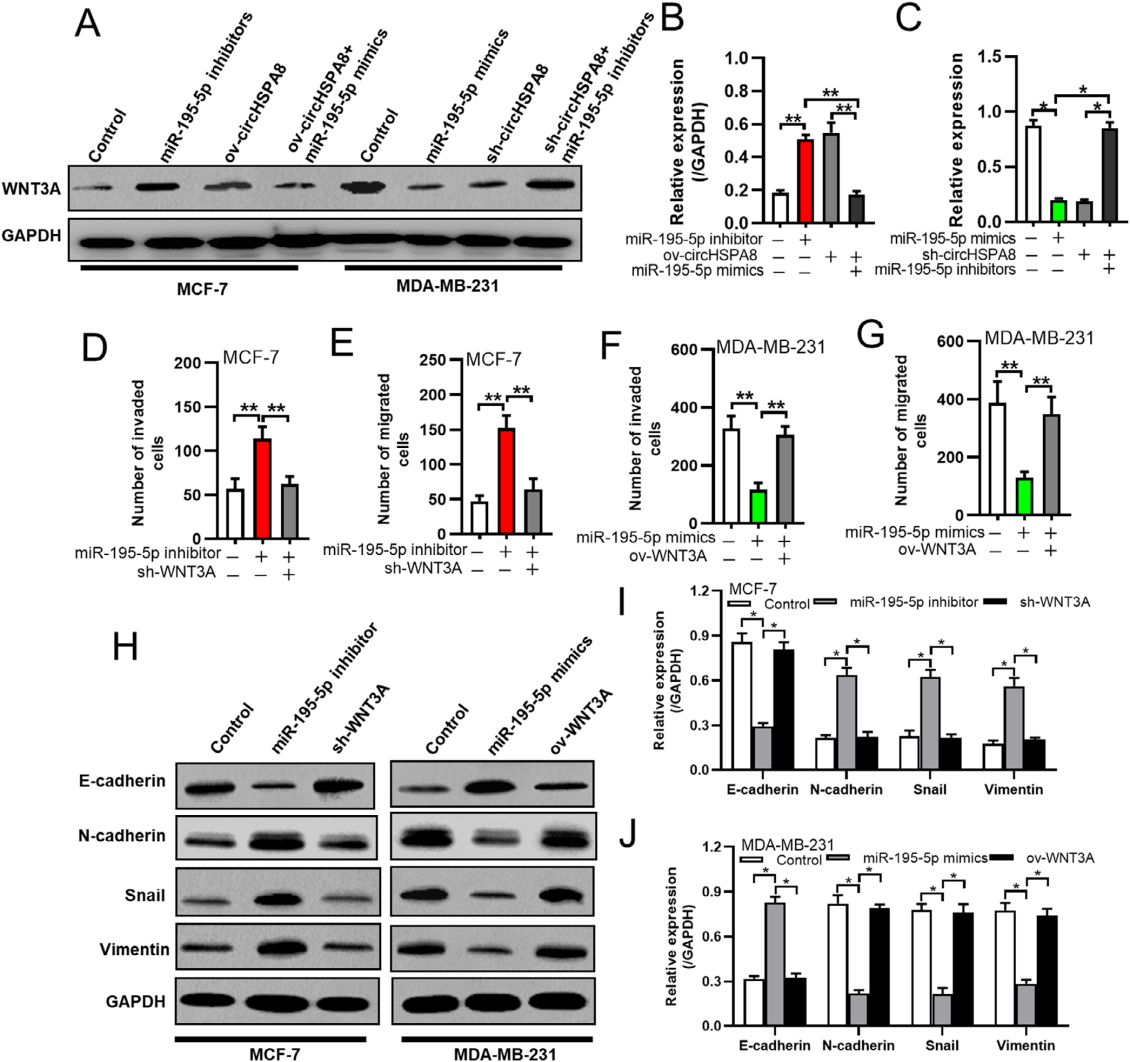
The circHSPA8‐miR‐195‐5p‐WNT3A axis promotes EMT and BC progression. (A–C) The expression of WNT3A was assessed via western blot analysis, followed by quantification using ImageJ software. The experimental conditions: MCF‐7 cells transfected with a miR‐195‐5p inhibitor, MCF‐7 cells with overexpressed circHSPA8, MCF‐7 cells co‐transfected with miR‐195‐5p mimics and a circHSPA8 overexpression plasmid, MDA‐MB‐231 cells transfected with miR‐195‐5p mimics, MDA‐MB‐231 cells with circHSPA8 knockdown, and MDA‐MB‐231 cells with circHSPA8 knockdown followed by transfection with a miR‐195‐5p inhibitor. (D and E) The invasiveness and migration of MCF‐7 cells, which were transfected with a miR‐195‐5p inhibitor or co‐transfected with sh‐WNT3A, were assessed using a transwell assay. (F and G) MDA‐MB‐231 cells were transfected with miR‐195‐5p mimics or co‐transfected with an overexpression WNT3A plasmid, and the invasiveness and migration of the cells were assessed using a transwell assay. (H–J) MCF‐7 cells were transfected with a miR‐195‐5p inhibitor or co‐transfected with sh‐WNT3A, while MDA‐MB‐231 cells were transfected with miR‐195‐5p mimics or co‐transfected with an overexpression WNT3A plasmid. Subsequently, the expression of epithelial‐mesenchymal transition (EMT)‐related proteins was assessed via Western blot analysis (H) and quantitatively analysed using ImageJ software (I‐J). *p < 0.05, **p < 0.01. Data are from three independent experiments.

To further validate this hypothesis, we transfected MCF‐7 cells with miR‐195‐5p inhibitors, circHSPA8 overexpression constructs, or a combination of circHSPA8 overexpression followed by miR‐195‐5p mimics. In MDA‐MB‐231 cells, we used miR‐195‐5p mimics and knocked down circHSPA8 expression, followed by transfection with miR‐195‐5p inhibitors. The results showed that, in MCF‐7 cells, both miR‐195‐5p inhibitors and circHSPA8 overexpression upregulated WNT3A expression, whereas miR‐195‐5p mimics inhibited WNT3A expression after circHSPA8 overexpression (Figure 6A,B). Similarly, in MDA‐MB‐231 cells, both miR‐195‐5p mimics and circHSPA8 knockdown downregulated WNT3A expression, whereas WNT3A expression was restored by miR‐195‐5p inhibitors after circHSPA8 knockdown (Figure 6A,C). These findings indicated that circHSPA8 regulates WNT3A expression in BC cells by sponging miR‐195‐5p.

*WNT3A* expression promotes EMT in tumour cells. Therefore, we knocked down *WNT3A* expression by transfecting MCF‐7 cells with miR‐195‐5p inhibitors and overexpressed *WNT3A* by transfecting MDA‐MB‐231 cells with miR‐195‐5p mimics. We then assessed the invasive and migratory abilities of the cells via western blotting assays to detect the expression levels of EMT‐related proteins. Our results showed that miR‐195‐5p inhibitors significantly promoted MCF‐7 cell invasion and migration, downregulating E‐cadherin and upregulating N‐cadherin, Snail, and Vimentin. This effect was nullified by WNT3A knockdown (Figure 6D,E,H,I). Conversely, miR‐195‐5p mimics significantly inhibited MDA‐MB‐231 cell invasion and migration, upregulating E‐cadherin and downregulating N‐cadherin, Snail, and Vimentin, which was reversed by WNT3A overexpression (Figure 6F,H,J). These results suggested that the circHSPA8‐miR‐195‐5p‐WNT3A axis promotes EMT and BC progression.

### 3.6 CircHSPA8 Enhances the Ability of BC Cells to Extravasate and Form Metastatic Foci In Vivo

**FIGURE 7:**
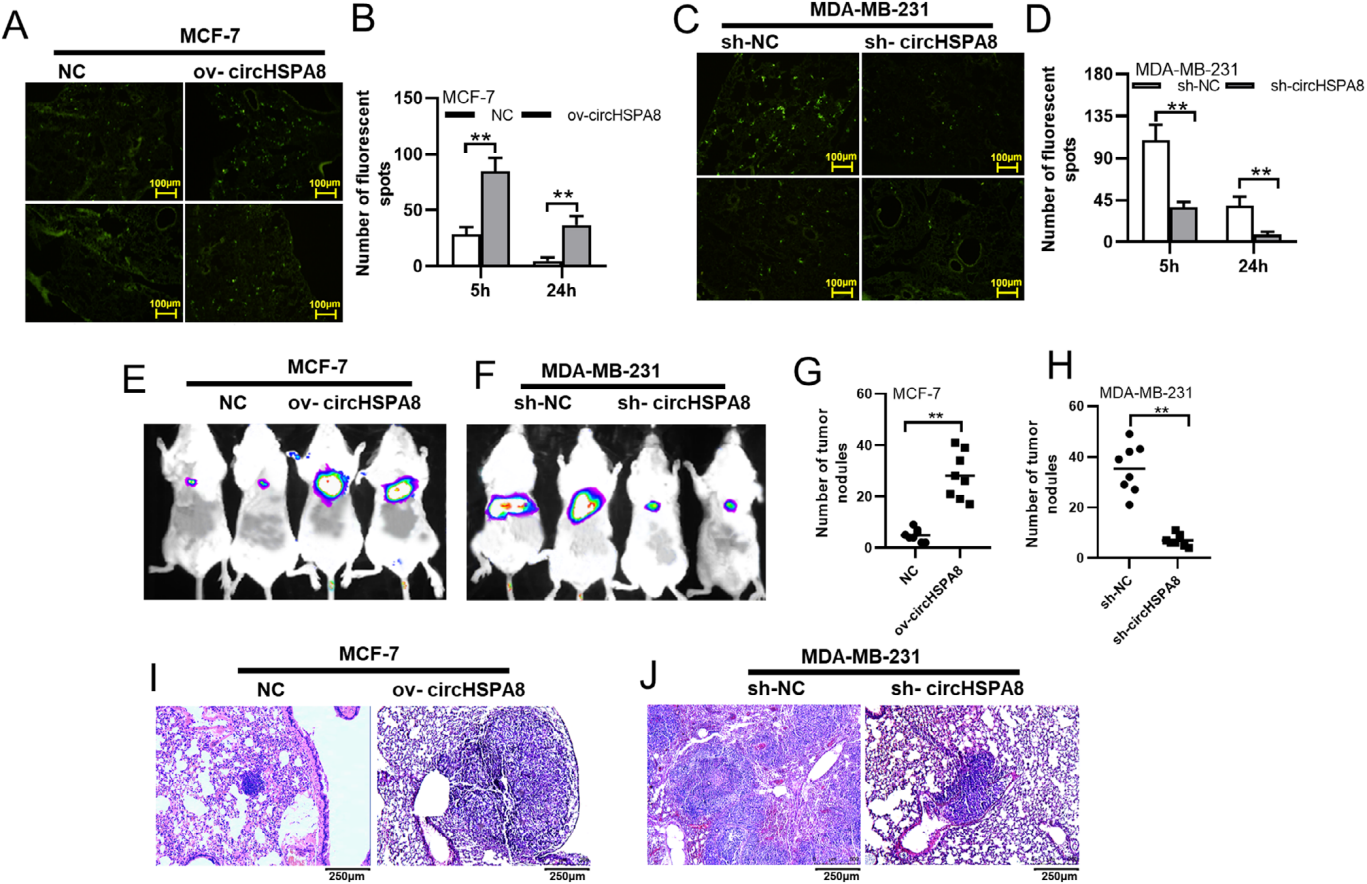
CircHSPA8 enhances the ability of BC cells to extravasate and form metastatic foci in vivo. (A–D). MCF‐7 and MDA‐MB‐231 cells were either left untransfected or transfected with the specified vectors, subsequently labelled with CFSE, and administered to NOD/SCID mice via tail vein injection. The mice (*n* = 5 per group) were euthanized at 5 h and 24 h post‐injection. CFSE‐labelled tumour cells within the frozen tissue sections were examined using fluorescence microscopy, and the fluorescent signals in the lung tissue sections were quantified. (E–H) MCF‐7 and MDA‐MB‐231 cells, either untransfected or transfected with the specified vectors, were administered to NOD/SCID mice through tail vein injection. Representative bioluminescent and photographic images of the NOD/SCID mice are presented in panels E and F. Four weeks post‐injection, the mice were euthanized using carbon dioxide asphyxiation, and the metastatic nodules present on the lung surface were quantified (G and H). (I and J) H&E staining was performed on paraffin‐embedded lung tissue sections, followed by microscopic examination and analysis of metastatic tumour lesions within the lung tissue. **p < 0.01. Data are from three independent experiments.

The occurrence of EMT in tumour cells promotes the metastasis of tumour foci to distant organs. We evaluated the effect of circHSPA8 on the metastatic ability of BC cells in vivo using a NOD‐SCID mouse lung metastasis model. MCF‐7 and MDA‐MB‐231 cells were labelled with CFSE and injected into NOD‐SCID mice via the tail vein. Mice were sacrificed at 5 and 24 h post‐injection, and frozen lung tissue sections were statistically analysed for fluorescent spots. The results showed that at 5 h post‐injection, MCF‐7 cells overexpressing circHSPA8 showed significantly increased retention in lung tissue (Figure 7A,B), whereas MDA‐MB‐231 cells with circHSPA8 knockdown showed significantly reduced retention in lung tissue (Figure 7C,D). At 24 h, fluorescent spots were observed only in the MCF‐7 cells overexpressing circHSPA8 and control MDA‐MB‐231 cells, whereas they were barely visible in the MCF‐7 control group and circHSPA8 knockdown MDA‐MB‐231 cells (Figure 7A–D). This suggests that circHSPA8 sufficiently enhances the metastatic potential of BC cells, enabling tumour cells to re‐extravasate into the lung tissue from the circulatory system.

To confirm the ability of BC cells to form metastatic foci after extravasation from circulation, we used in vivo imaging to observe the formation of metastatic foci in mouse lung tissues 4 weeks after inoculation with luciferase‐labelled cells. Strong signals were observed in MCF‐7 cells overexpressing circHSPA8 and control MDA‐MB‐231 cells, whereas weaker signals were observed in the other groups (Figure 7E,F). Post‐mortem statistical analysis of lung tissue showed a significant increase in lung metastases in circHSPA8‐overexpressing MCF‐7 cells compared to controls, and a significant decrease in lung metastases in circHSPA8‐knockdown MDA‐MB‐231 cells compared to controls(Figure 7G,H). HE staining of lung tissue sections further confirmed these results (Figure 7I,J). These findings indicated that circHSPA8 enhanced the ability of BC cells to extravasate and form metastatic foci in vivo.

## 4 Discussion

The biogenesis of circRNAs is dependent on alternative splicing site selection and back‐splicing circularization, regulated by the transcription of circRNA‐producing RNA molecules and the post‐transcriptional circularization of various cis/trans‐regulating factors, including RNA‐binding proteins such as adenosine deaminase 1 acting on RNA (ADAR1), NF90, and core spliceosomal components [13, 14, 15]. CircRNAs also contribute to tumorigenesis and the malignant biological properties of cancerous cells [7, 16, 17, 18]. Hundreds of circRNAs are upregulated or downregulated during human EMT, suggesting that circRNAs may play a crucial role in EMT‐induced biological behaviours, such as migration, invasion, and cancer metastasis [8, 19, 20]. A reduction in the abundance of certain circRNA molecules in colorectal cancer cell lines and a correlation between the dysfunction of circRNA abundance and proliferation have been reported previously [21, 22]. A recent study showed that reducing circPVT1 levels in proliferating fibroblasts could trigger apoptosis and senescence [23]. Moreover, circRNAs have also been implicated in the pathogenesis of acute promyelocytic leukaemia (APL), and the most common chromosomal translocation of PML/RARa in patients with APL leads to the abnormal formation of cancer‐specific circRNAs that promote cancer cell survival [24].

Competing endogenous RNAs (ceRNAs) can inhibit the biological effects of miRNAs by acting as sponges for miRNAs through their specific interaction with miRNAs, leading to the upregulation of target genes of miRNAs and alteration of the biological phenotype [25, 26, 27]. Profile changes in ceRNA abundance can regulate the target degeneration of miRNAs for downstream genes. Several circRNAs can function as miRNA sponges. For example, in hepatocellular carcinoma, circRNA _104348 absorbs free miR‐187‐3p by binding to specific sites on miR‐187‐3p to repress its biological activity, which promotes cell proliferation and invasion [28]. Several other circRNAs in mammals are considered potential miRNA sponges. For example, circHIPK2 may act as a sponge for miR‐124 to regulate astrocyte activation via autophagy and endoplasmic reticulum (ER) stress [29]. CircBIRC6 modulates human ES pluripotency and differentiation by sequestering miR‐34a and miR‐145 [30]. Although many miRNA‐binding sites have been predicted in various circRNA datasets, the regulatory roles of these circRNAs on their potentially bound miRNAs are largely unexplored. It should be noted that the majority of circRNAs in mammals are expressed at low levels and rarely contain multiple binding sites for the same miRNAs; thus, it seems unlikely that many circRNAs can function as miRNA sponges. In malignant tumours, circRNAs serve as sponges for inhibiting miRNAs. In hepatocellular carcinoma, the knockout of ADAR1, a crucial regulator in circRNA formation, interferes with complementary pairs of RNA intron sequences and circularisation of circRNAs, leading to increased proliferation in HepG2 and SK‐Hep1 cell lines [31, 32]. These studies have demonstrated the transcription‐regulating function of ceRNAs as molecular sponges across many biological processes, including autophagy, ER stress, differentiation, and tumour biology.

In this study, we observed increased expression of circHSPA8 in previously shared raw data related to non‐coding RNA profiles between BC and non‐cancerous tissues. We predicted and verified the downstream target miRNAs and genes affected by the increased expression of circHSPA8. The biogenesis of circHSPA8 involves the back‐splicing of exons in the HSPA8 gene, resulting in a covalently closed loop. This unique structure of circHSPA8 allows it to evade degradation by exonucleases, thus stabilising the molecule within cells. While no study has specifically examined the expression levels of circHSPA8 in relation to the upregulation of linear HSPA8 in breast cancer, it is plausible that the regulation and function of circHSPA8 expression are intricately connected to the HSPA8 gene. HSPA8 is a member of the mammalian HSP70 family, a group of proteins that play an important role in guiding correct protein folding, maintaining protein stability, and promoting cell survival under various stress conditions. As a molecular chaperone, HSPA8 cooperates with protein transport complexes to transport various proteins from the cytoplasm to intracellular organelles, such as the ER and mitochondria, where they perform their corresponding functions [10, 33, 34, 35]. HSPA8 has been implicated in various cancer types due to its involvement in maintaining cellular homeostasis under stress conditions. It has been shown to interact with multiple signalling pathways that regulate cell survival, apoptosis, and metastasis, all of which are critical in cancer development [36]. For instance, in hepatocellular carcinoma (HCC), HSPA8 functions as a co‐activator of the transcription factor ETV4, leading to the upregulation of PHLDA2, thereby facilitating the progression of liver cancer [37]. In cases of liver cancer linked to viral hepatitis, HBx‐induced HSPA8 enhances HBV replication and inhibits ferroptosis, thereby facilitating the progression of liver cancer [38]. Recent research indicates that HSPA8 enhances the progression of BRAF V600E colorectal cancer by activating Wnt/β‐Catenin signalling through CMA‐mediated degradation of CAV1 [39]. In BC, HSPA8 is significantly upregulated and is closely associated with prognosis and immune infiltration [10]. However, the role of HSPA8 in BC and its detailed mechanism of action remain unclear.

In the present study, we found that elevated expression levels of circHSPA8 in BC were closely associated with poor prognosis and lymph node metastasis. In the investigation of breast cancer metastasis, numerous reports suggest that MCF‐7 cells serve as a model for low invasive epithelial cells, typically characterised by elevated levels of E‐cadherin and reduced levels of N‐cadherin, vimentin, and Snail. Conversely, MDA‐MB‐231 cells are highly invasive mesenchymal cells, displaying an inverse expression pattern [40, 41, 42]. This distinction is intricately linked to the epithelial–mesenchymal transition (EMT) process, wherein epithelial cells lose intercellular adhesion and acquire migratory and invasive capabilities [43]. The differential expression observed between these cell types aligns with the classical model of breast cancer cell invasion, wherein low invasive cells (such as MCF‐7) predominantly exhibit epithelial traits, while highly invasive cells (such as MDA‐MB‐231) demonstrate mesenchymal characteristics [44]. This phenomenon is of considerable importance for elucidating the mechanisms underlying breast cancer invasion and metastasis. Interestingly, circHSPA8 expression levels were significantly higher in the highly invasive MDA‐MB‐231 cells than in the less invasive MCF‐7 cells. In vitro cell experiments confirmed that circHSPA8 promotes EMT in BC cells by acting as a sponge to absorb the target miR‐195‐5p and upregulates the expression of downstream genes. In vivo experiments demonstrated that circHSPA8 enhances the metastatic potential of breast cancer cells in murine models.

Furthermore, we found increased expression of WNT3A in the MCF‐7 cell line after overexpression of circHSPA8, and decreased expression of WNT3A in MD‐MB‐231 cells after knockdown of circHSPA8. Previous studies have reported that WNT3A induces transcriptome changes characterised by the activation of the canonical WNT/β‐catenin signalling pathway in triple‐negative BC cells. Activation of the canonical WNT pathway leads to enhanced EMT in cancerous cells, which has been proven to be related to invasion and metastasis. Therefore, increased invasion and metastasis could be explained by the higher expression of WNT3A following overexpression of circHSPA8. Overproduction and aberrant release of WNT3A in BC cells may affect adjacent cancerous cells in a paracrine manner, activating the canonical WNT pathway. We propose that dysregulation of WNT3A expression in breast cancer leads to more aggressive behaviour, independent of upstream miR‐195‐5p repression after circHSPA8 overexpression.

In summary, we identified a novel circRNA molecule, abnormally expressed in highly invasive and metastatic BC cells and in the majority of breast carcinoma tissues from a patient cohort. Abnormal transcription of circHSPA8 leads to the overexpression of oncogenes by acting as a sponge to repress the regulatory effect of microRNA‐195‐5p. The discovery of circHSPA8 adds a new dimension to our understanding of HSPA8's function in cancer biology. Given the potential of circHSPA8's oncogenic properties, further research is needed to fully elucidate the mechanisms through which circHSPA8 contributes to cancer progression. Our findings provide new insights into the dysregulation of non‐coding RNA profiles in the tumour biology of BC, especially in breast carcinomas with more rapid growth and highly metastatic tendencies.

## Author Contributions

Zhuoying Han*: data curation (equal), investigation (equal), methodology (equal), software (equal). Xiaojuan Yu*: data curation (equal), investigation (equal), methodology (equal), writing – review and editing (equal). Chenlong Wang*: data curation (equal), methodology (equal). **Xiaoyu Song:** investigation (equal), methodology (equal), software (equal). **Xiaomin Zhong:** data curation (equal), formal analysis (equal), investigation (equal), writing – original draft (equal). **Renhua Guo:** funding acquisition (equal), project administration (equal), supervision (equal), writing – review and editing (equal). **Weiyong Yu:** conceptualization (equal), data curation (equal), funding acquisition (equal), writing – review and editing (equal). **Chao Luo:** conceptualization (equal), funding acquisition (equal), investigation (equal), project administration (equal), supervision (equal), writing – original draft (equal), writing – review and editing (equal).

## Conflicts of Interest

The authors declare no conflicts of interest.

## Data Availability

The authors have nothing to report.
