# Correction to “Circular RNA circHSPA8 Aggravates Metastasis by Acting as a Competitive Inhibitor of miR‐195‐5p to Upregulate WNT3A Expression in Breast Cancer”

**DOI:** 10.1111/jcmm.70673

**Published:** 2025-07-15

Z. Han, X. Yu, C. Wang, et al., “Circular RNA circHSPA8 Aggravates Metastasis by Acting as a Competitive Inhibitor of miR‐195‐5p to Upregulate WNT3A Expression in Breast Cancer” *Journal of Cellular and Molecular Medicine* 29 (2025):e70499, https://doi.org/10.1111/jcmm.70499.

In the article, the figure 2 panels M and O contain errors. The correct Figure 2M and 2O are shown below.

The authors confirmed that these corrections do not affect the conclusion and overall findings of the study.

We apologize for these errors.

**Figure:**
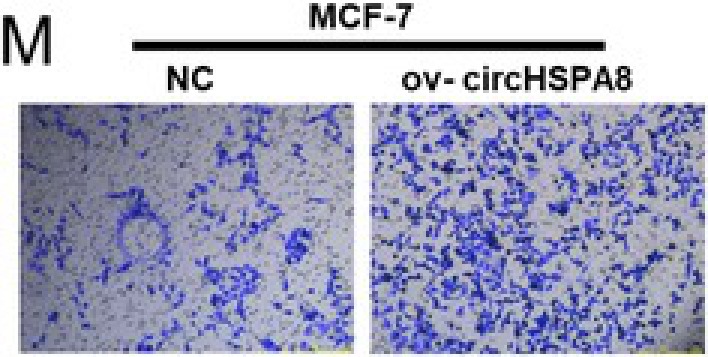
Figure 2M

**Figure:**
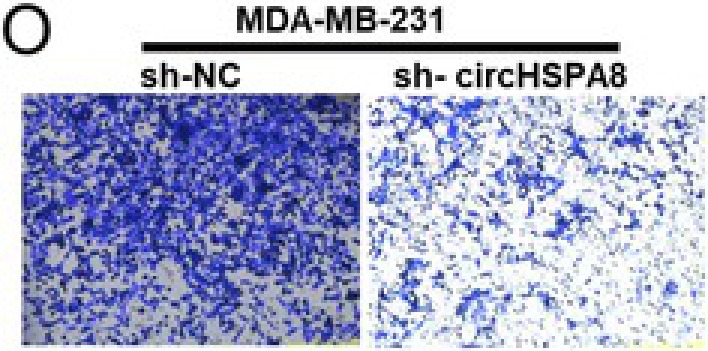
Figure 2O

